# Jinshui Liujun Decoction attenuates cigarette smoke–induced small airway remodeling via the TGF-β/Smad2/CFTR Axis

**DOI:** 10.1016/j.jphyss.2026.100095

**Published:** 2026-08-20

**Authors:** Yu Liu, Wenjuan Xie, Qijun Zhou, Guo Ouyang

**Affiliations:** a Department of Pulmonology, Hunan Academy of Traditional Chinese Medicine Affiliated Hospital, Changsha, Hunan Province 410006, PR China; b Department of Emergency, The First Hospital of Hunan University of Chinese Medicine, Changsha, Hunan Province 410007, PR China; c Hunan University of Chinese Medicine, Changsha, Hunan Province 410208, PR China

**Keywords:** Chronic obstructive pulmonary disease, Small airway remodeling, Epithelial–mesenchymal transition, CFTR, Jinshui Liujun Decoction

## Abstract

Small airway remodeling is a key pathological feature of chronic obstructive pulmonary disease (COPD), and epithelial—mesenchymal transition (EMT) is among the central mechanisms driving this remodeling process. Jinshui Liujun Decoction (JSLJD), a traditional Chinese herbal formula, has shown therapeutic potential in respiratory diseases, but its effects on COPD-associated small airway remodeling remain unclear. In this study, JSLJD significantly reduced collagen accumulation, airway wall thickening, and α-SMA expression in the lungs of cigarette smoke (CS)-exposed mice. In vivo and in vitro, JSLJD inhibited EMT, as reflected by elevated E-cadherin expression and reduced vimentin, fibronectin, and Snail expression. Mechanistically, JSLJD inhibited TGF-β/Smad2 activation and restored cystic fibrosis transmembrane conductance regulator (CFTR) expression. Pharmacological blockade of CFTR or activation of TGF-β abrogated the anti-EMT effects of JSLJD. In conclusion, JSLJD alleviates CS-induced small airway remodeling by suppressing EMT via the inhibition of the TGF-β/Smad2 pathway and the recovery of CFTR function, highlighting its promise as a prospective therapy for COPD.

## Highlights

• JSLJD alleviated small airway structural remodeling and fibrotic alterations in cigarette smoke–induced COPD models.
• JSLJD inhibited epithelial—mesenchymal transition (EMT) in mouse lungs subjected to cigarette smoke exposure.
• By inhibiting TGF-β/Smad2 signaling and restoring CFTR expression, JSLJD suppressed EMT and small airway remodeling.

## Introduction

Chronic obstructive pulmonary disease (COPD) remains a major global health burden and is characterized by long-term airflow obstruction and chronic respiratory manifestations [1]. Small airway remodeling is recognized as a central pathological hallmark of COPD progression, and it manifests as epithelial–mesenchymal transition (EMT), peribronchiolar fibrosis, and smooth muscle hypertrophy [2]. Among these mechanisms, EMT in airway epithelial cells is considered a major contributor to structural alterations, linking chronic inflammation to irreversible airflow obstruction [2], [3]. Despite the availability of inhaled corticosteroids and bronchodilators that reduce inflammation and improve airway patency, none of the current therapies effectively reverses small airway remodeling, underscoring the pressing demand for new treatment approaches [4], [5].

Jinshui Liujun Decoction (JSLJD), a classical traditional Chinese medicine (TCM) prescription, has long been applied in the management of chronic respiratory disorders [6]. It exhibits anti-inflammatory, antioxidative, and immunoregulatory properties, with demonstrated clinical benefits in alleviating respiratory symptoms and improving lung function [7]. Our previous studies have shown that JSLJD reduces mucus hypersecretion in COPD animal models [8]. To date, no studies have reported the effects of JSLJD on EMT and small airway remodeling in models of COPD.

Cystic fibrosis transmembrane conductance regulator (CFTR), which functions as a cAMP-regulated chloride channel, plays a vital role in maintaining ion and fluid homeostasis in the airway epithelium [9]. Cigarette smoke (CS), the major etiological factor contributing to COPD, suppresses CFTR expression and function, thereby exacerbating mucus stasis, epithelial injury, and airway fibrosis [10]. CFTR deficiency is implicated in the progression of small airway remodeling [11]. Our previous work demonstrated that JSLJD significantly increases CFTR mRNA and protein expression in murine lung tissue [12], suggesting a potential role for CFTR restoration in mediating the therapeutic effects of JSLJD. However, whether JSLJD alleviates CS-induced small airway remodeling by upregulating CFTR expression remains to be elucidated.

Transforming growth factor-β (TGF-β) acts as a pleiotropic cytokine involved in cellular growth control, phenotypic differentiation, and tissue remodeling [13]. In pulmonary diseases such as cystic fibrosis, COPD, and asthma, the persistent activation of TGF-β signaling contributes to epithelial injury, small airway remodeling, and fibrosis [14]. Among its downstream pathways, the canonical TGF-β/Smad2/3 cascade plays a central role in driving EMT, a process during which epithelial cells gradually lose polarity and gain mesenchymal traits [15]. Accumulating data indicate that TGF-β signaling not only promotes EMT in airway epithelial cells but also suppresses CFTR expression, thereby exacerbating mucus hypersecretion and impairing epithelial ion transport [16], [17]. The inhibition of TGF-β/Smad2 activation has been shown to restore CFTR function and attenuate EMT-associated small airway remodeling, highlighting this pathway as a potential therapeutic target for chronic airway diseases [18].

Overall, we hypothesize that JSLJD attenuates CS-induced small airway remodeling by suppressing TGF-β/Smad2 signaling and restoring CFTR expression, thereby inhibiting EMT in bronchial epithelial cells. This study integrates in vivo and in vitro approaches to clarify the mechanistic basis of JSLJD, aiming to provide novel insights into its therapeutic potential for COPD.

## Methods

### Animal Model and Grouping

Male C57BL/6 J mice aged 6–8 weeks and weighing 20–22 g were obtained from the Shanghai SLAC Laboratory Animal Center (China). All the animals were kept in SPF facilities and provided with food and water ad libitum. The animals were randomly allocated into four groups (six mice per group): Control, CS, CS + L-JSLJD, and CS + H-JSLJD. At 8 weeks of age, the mice in the CS and CS + JSLJD groups were exposed to CS in a whole-body exposure chamber (20 cigarettes/time, 1 h/time, twice daily, 6 days/week) for 12 consecutive weeks. The Control group was exposed to room air under identical conditions for the same duration. The CS + L-JSLJD and CS + H-JSLJD groups received JSLJD at doses of 7.41 and 14.82 g/kg/day, respectively, by oral gavage 30 min before the first CS exposure each day. The Control and CS groups were given an equivalent volume of physiological saline. The mice were euthanized 24 h after the last exposure, and lung tissues were harvested for further examination. All animal procedures were approved by the Institutional Animal Care and Use Committee of the Hunan Academy of Traditional Chinese Medicine Affiliated Hospital.

### Preparation of JSLJD

JSLJD extract was prepared following a previously published protocol [8]. It was prepared from authenticated herbal components according to the traditional formula recorded in Jingyue Quanshu. The classical composition comprises Rehmannia glutinosa (15 g), Angelica sinensis (7.5 g), Pericarpium Citri Reticulatae (5.5 g), Pinellia ternata (7.5 g), Poria cocos (7.5 g), honey-fried Glycyrrhiza (4 g), and ginger (10 g). All the herbal materials used were purchased from the Pharmacy Department of the Affiliated Hospital of the Hunan Academy of Traditional Chinese Medicine. The medicinal materials were authenticated according to the standards of the Chinese Pharmacopoeia (2020 edition) by qualified specialists in traditional Chinese medicine identification on the basis of macroscopic characteristics. The herbs were soaked in distilled water (1:10, w/v) for 30 min and decocted twice for 1.5 h and 1 h, respectively. The combined filtrates were filtered and concentrated under reduced pressure to yield a crude drug concentration of 1 g/mL. For the animal experiments, the concentrated extract was freshly diluted with sterile saline to the required dose immediately before oral gavage.

### Masson’s Trichrome Staining

Lung specimens were fixed in 4% paraformaldehyde, embedded in paraffin, and sectioned at a thickness of 4 μm. Masson’s trichrome staining (Solarbio, China) was performed to evaluate collagen deposition. Briefly, the sections were immersed in hematoxylin for nuclear staining, followed by incubation with Ponceau-acid fuchsin solution to stain the cytoplasm and muscle fibers. Following differentiation in phosphomolybdic acid, the sections were stained with aniline blue to highlight the collagen fibers. The samples were then dehydrated, cleared, and mounted using neutral resin. Quantitative morphometric analysis was performed using ImageJ software. Airway wall thickness and collagen-positive areas were measured in randomly selected complete small airway cross-sections. For each mouse, five small airways (diameter 100–200 μm and long-to-short axis ratio ≤1.5) were analyzed. Airway wall thickness was quantified by measuring the airway wall area normalized to the basement membrane perimeter, and it was expressed as μm. Collagen deposition was quantified as the percentage of the collagen-positive area within the airway wall.

### Immunofluorescence Staining

Frozen lung Section (10 μm) were prepared from OCT-embedded lung tissues and fixed with 4% paraformaldehyde. After being washed with PBS, the sections were blocked with 5% BSA for 30 min at room temperature and incubated overnight at 4 °C with primary antibodies against α-SMA (CST, #19245, 1:400), E-cadherin (CST, #3195, 1:1600), and vimentin (CST, #5741, 1:100). After the samples were washed, they were incubated with goat anti-rabbit IgG H&L (Alexa Fluor® 488) (Abcam, ab150077, 1:500) and goat anti-rabbit IgG H&L (Alexa Fluor® 555) (Abcam, ab150078, 1:500) for 1 h at room temperature, after which they were counterstained with DAPI. Sections were mounted with antifade medium and imaged using a fluorescence microscope (Olympus). The α-SMA-positive area was calculated as the percentage of the α-SMA-positive area within the total airway wall area.

### Western blot Analysis

Mouse lung tissues and 16HBE cells were lysed in RIPA buffer (Beyotime, China) supplemented with protease/phosphatase inhibitors, after which the protein concentration was determined via a BCA assay (Thermo, USA). Proteins were separated by SDS—PAGE and transferred to PVDF membranes (Millipore, IPVH00010, USA). After blocking, the membranes were incubated overnight at 4 °C with the following primary antibodies: CFTR (Proteintech, 66928–1-Ig, 1:5000), p-Smad2 (Cell Signaling Technology, #3108, 1:1000), Smad2 (Proteintech, 12570–1-AP, 1:2000), TGF-β1 (Abcam, ab215715, 1:1000), α-SMA (Abcam, ab7817, 1 µg/mL), Collagen I (Abcam, ab316222, 1:2000), Collagen III (Proteintech, 68320–1-Ig, 1:5000), E-cadherin (Cell Signaling Technology, #3195, 1:1000), fibronectin (Abcam, ab2413, 1 µg/mL), vimentin (Abcam, ab92547, 1:2000), Snail (Cell Signaling Technology, #3879, 1:1000), and β-actin (Abcam, ab8227, 1:3000). Blots were developed using HRP-conjugated secondary antibodies (Proteintech, SA00001–1 or SA00001–2, 1:5000) and visualized using enhanced chemiluminescence (ECL; Thermo, 32106, USA).

### Quantitative Real-Time Polymerase Chain Reaction (qPCR)

Total RNA was extracted using TRIzol reagent (Invitrogen, 15596026, USA), and cDNA synthesis was conducted using a reverse transcription kit (Takara, RR047A, Japan). qPCR was performed using SYBR Green Master Mix (Applied Biosystems, A25742, USA). β-actin was used as the internal reference. The 2^−ΔΔCt method was used to calculate the relative expression levels. The primer sequences are listed in Table 1.

**Table 1:** Sequences of primers used in this study.

Gene	Sequence (5′−3′)
Mouse β-actin	F: AGAGGGAAATCGTGCGTGAC
	R: CAATAGTGATGACCTGGCCGT
Mouse Collagen I	F: CCTCAGGGTATTGCTGGACAAC R: CAGAAGGACCTTGTTTGCCAGG
Mouse Collagen III	F: GACCAAAAGGTGATGCTGGACAG R: CAAGACCTCGTGCTCCAGTTAG
Mouse TGF-β	F: TGATACGCCTGAGTGGCTGTCT R: CACAAGAGCAGTGAGCGCTGAA
Mouse Smad2	F: CCAACTGTAACCAGAGATACGGC R: AACCCTGGTTGACAGACTGAGC
Mouse CFTR	F: CCATCAGCAAGCTGAAAGCAGG R: GTAGGGTTGTAATGCCGAGACG
Human β-actin	F: CCCTGGAGAAGAGCTACGAG R: CGTACAGGTCTTTGCGGATG
Human TGF-β	F: TACCTGAACCCGTGTTGCTCTC R: GTTGCTGAGGTATCGCCAGGAA
Human Smad2	F: GGGTTTTGAAGCCGTCTATCAGC R: CCAACCACTGTAGAGGTCCATTC
Human CFTR	F: GGAGAGCATACCAGCAGTGACT R: TTCCAAGGAGCCACAGCACAAC
Human α-SMA	F: CTATGCCTCTGGACGCACAACT R: CAGATCCAGACGCATGATGGCA
Human Collagen I	F: GATTCCCTGGACCTAAAGGTGC R: AGCCTCTCCATCTTTGCCAGCA

### Preparation of Cigarette Smoke Extract (CSE)

CSE was freshly prepared by bubbling smoke from one commercial cigarette (Hongtashan, Yunnan Tobacco, China; 11 mg tar, 0.9 mg nicotine, 12 mg CO) into 10 mL of serum-free RPMI−1640 medium using a vacuum pump at a constant rate (1 cigarette/5 min). The extract was passed through a 0.22 µm filter (Millipore, USA) and standardized by measuring the absorbance at 320 nm (OD320 = 0.8) with a 1 cm path-length cuvette. The CSE was used within 30 min to avoid oxidation and was subsequently diluted to 2.5% (v/v) in serum-free medium for cell stimulation.

### Preparation of Drug-Containing Serum

Male Sprague–Dawley rats (approximately 200 ± 20 g, two months old) were acclimated for one week under standard conditions and randomly assigned to a JSLJD group or a blank Control group. The JSLJD group was given JSLJD (10.26 g/kg/day, once daily) by oral gavage for 7 consecutive days. The Control animals received an equivalent volume of saline. Two hours after the final administration, the rats were anesthetized with 2–3% isoflurane in oxygen, and blood was aseptically collected from the abdominal aorta. Blood samples were left at room temperature for 2 h and then centrifuged at 3000 rpm for 15 min to harvest the serum. The serum was heat-treated at 56 °C for 30 min, sterile-filtered (0.22 μm), divided into aliquots, and stored at −20 °C. For the cell experiments, the JSLJD-containing serum was diluted in culture medium to yield a 10% working concentration.

### Cell Culture and Treatment

16HBE cells were maintained in RPMI−1640 medium (Gibco) supplemented with 10% fetal bovine serum and 1% penicillin—streptomycin, and they were incubated at 37 °C in a humidified atmosphere with 5% CO₂. The cells were divided into five groups: Control, CSE, CSE + JSLJD serum, CSE + JSLJD serum + TGF-β activator (SRI−011381; MedChemExpress, HY−13901, USA), and CSE + JSLJD serum + CFTR inhibitor (steviol; Sigma—Aldrich, S3676, USA). The cells were pretreated with 10% serum and/or activators/inhibitors for 2 h, followed by stimulation with 2.5% CSE. After 48 h, the cells were harvested for RNA and protein analyses.

### Statistical Analysis

The data are expressed as the mean ± SD. Normality was assessed using the Shapiro–Wilk test. For comparisons among multiple groups, a one-way ANOVA with Tukey’s multiple-comparisons test was used for normally distributed data. A two-sided P value of < 0.05 was considered to indicate statistical significance. Analyses were performed in GraphPad Prism version 10.1.2 (USA). For histological and morphometric analyses, image quantification was performed by investigators who were blinded to group allocation to minimize potential bias.

## Results

### JSLJD attenuated CS-induced small airway remodeling in mice

**Fig. 1:**
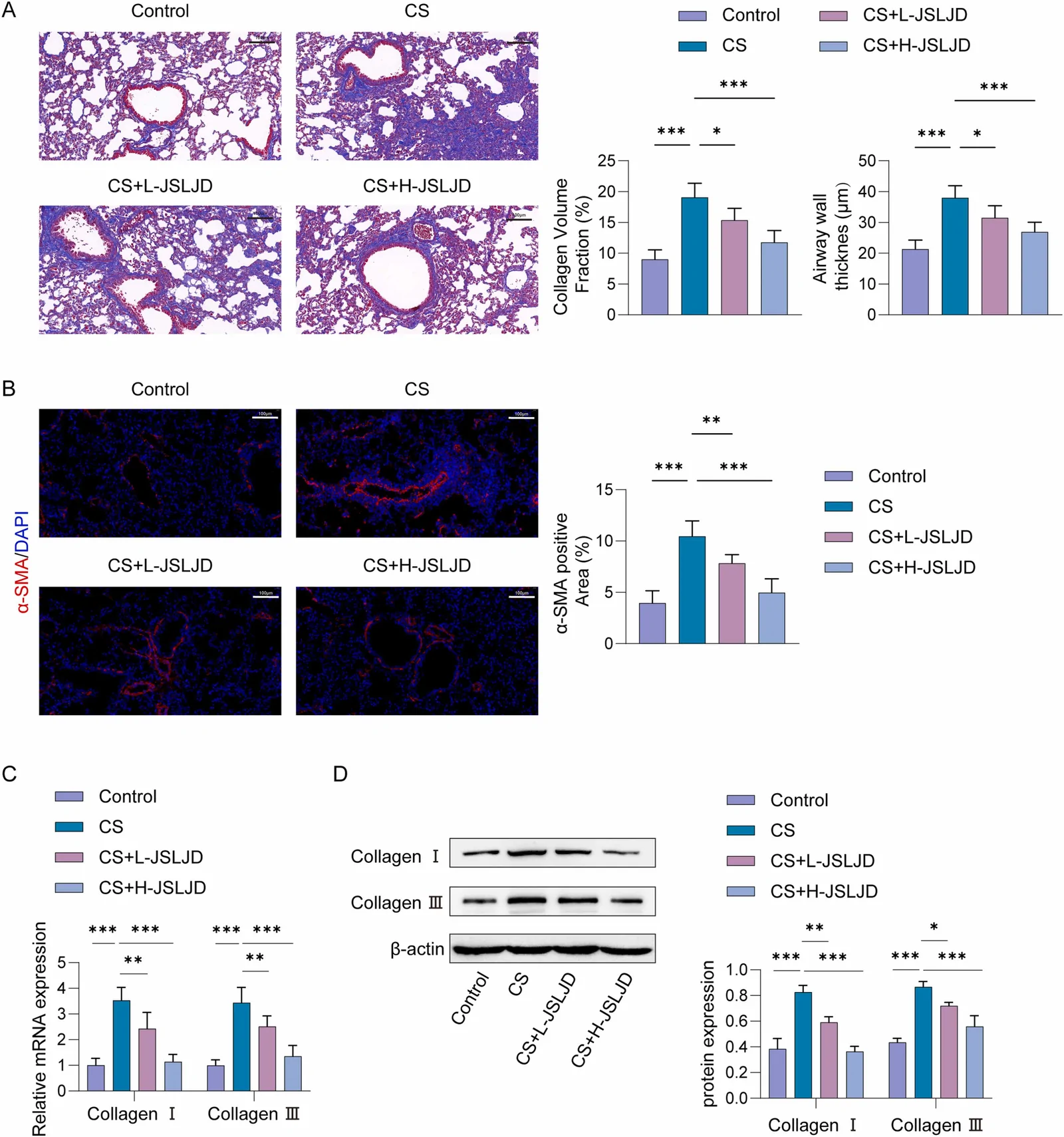
JSLJD attenuated CS-induced small airway remodeling in mice. (A) Representative Masson’s trichrome staining images and quantitative analysis of airway wall thickness and collagen-positive areas in mouse lungs. (B) Representative α-SMA immunofluorescence images and quantitative analysis of the α-SMA-positive area in peribronchiolar regions. (C) qPCR analysis of Collagen I and Collagen III mRNA levels in lung tissues. (D) Western blot detection of Collagen I and Collagen III protein expression in mouse lungs. The data are presented as the mean ± SD (n = 6 mice per group). Statistical comparisons among the Control, CS, CS + L-JSLJD, and CS + H-JSLJD groups were performed using a one-way ANOVA followed by Tukey’s multiple comparisons test. *P < 0.05, **P < 0.01, and ***P < 0.001 indicate significant differences between the indicated groups.

Small airway remodeling is a key pathological feature in the development of COPD. We first assessed the impact of JSLJD on CS-induced small airway remodeling. Masson’s trichrome staining combined with quantitative morphometric analysis revealed pronounced collagen accumulation and airway wall thickening in the CS group, as evidenced by increased collagen-positive areas and airway wall thickness (Fig. 1A). Following low-dose JSLJD treatment, the airway walls became visibly thinner, and collagen deposition was markedly reduced, whereas high-dose treatment almost completely suppressed CS-induced airway remodeling (Fig. 1A). Immunofluorescence staining revealed intense α-SMA expression in the peribronchiolar region of the CS-exposed lungs, which substantially decreased after JSLJD administration (Fig. 1B). Furthermore, qPCR and Western blot analyses revealed that CS exposure significantly upregulated Collagen I and Collagen III expression, whereas JSLJD effectively reversed these changes (Fig. 1C–D). These findings indicate that JSLJD ameliorates CS-induced small airway remodeling in mice.

### JSLJD inhibited CS-induced EMT in mouse lung tissues

**Fig. 2:**
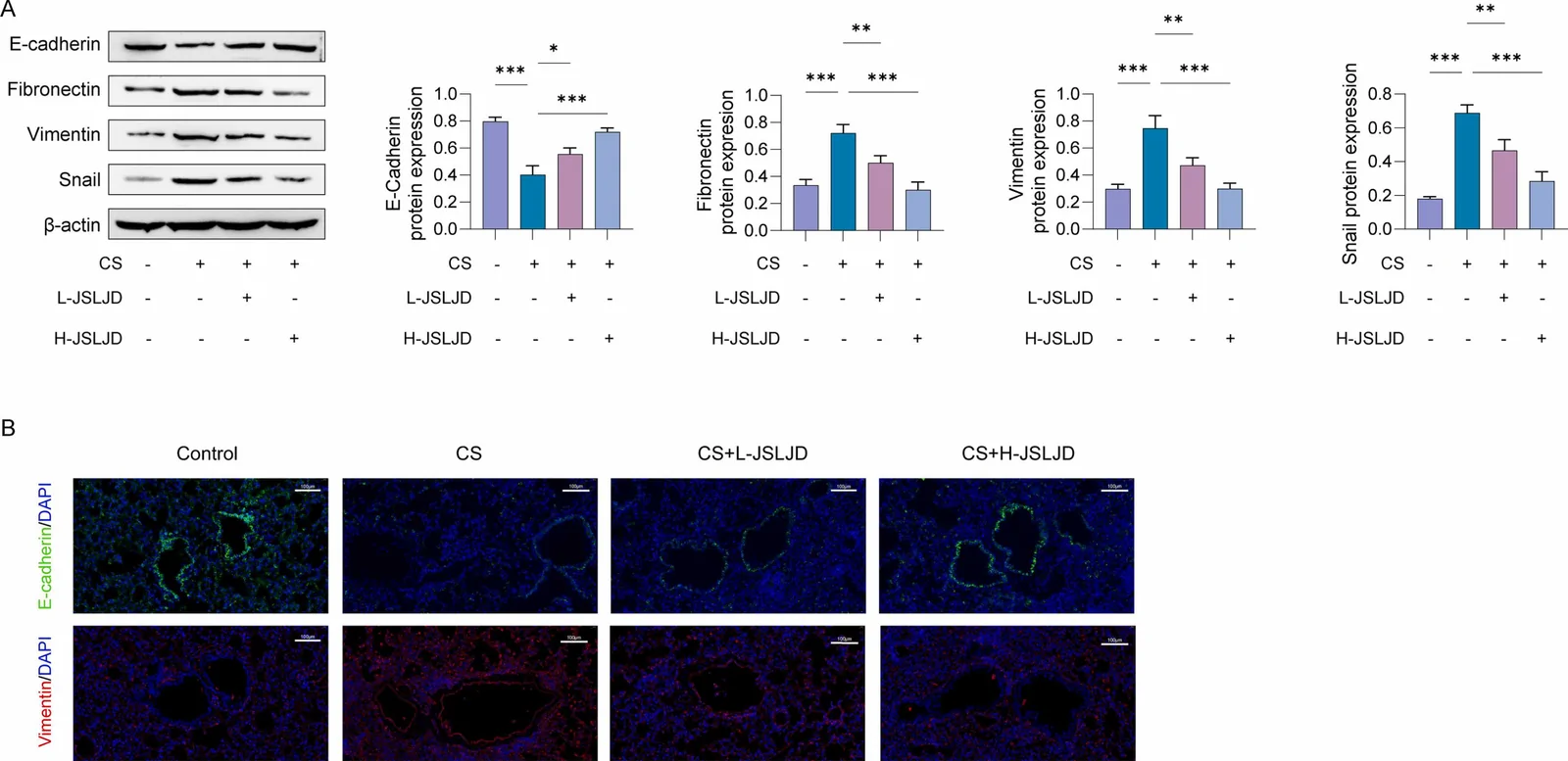
JSLJD inhibited CS-induced epithelial–mesenchymal transition in mouse lungs. (A) Western blot detection of the EMT-related proteins E-cadherin, fibronectin, vimentin, and Snail in lung tissues. (B) Immunofluorescence staining for E-cadherin and vimentin in mouse lung sections. The data are presented as the mean ± SD (n = 6 mice per group). Statistical comparisons among the Control, CS, CS + L-JSLJD, and CS + H-JSLJD groups were performed using a one-way ANOVA followed by Tukey’s multiple comparisons test. *P < 0.05, **P < 0.01, and ***P < 0.001 indicate significant differences between the indicated groups.

EMT is a critical process contributing to small airway remodeling in patients with COPD. We next examined whether JSLJD mitigates CS-induced EMT in vivo. Western blot analysis revealed that CS exposure markedly upregulated the expression of mesenchymal proteins (fibronectin, vimentin, and Snail) and downregulated the expression of the epithelial protein E-cadherin. Treatment with both low and high doses of JSLJD reversed these alterations in a dose-dependent manner, restoring E-cadherin expression and reducing mesenchymal marker levels (Fig. 2A). Immunofluorescence staining further confirmed these findings: CS exposure led to decreased E-cadherin expression and increased vimentin fluorescence, both of which were substantially normalized after JSLJD administration (Fig. 2B). Collectively, these data indicate that JSLJD effectively suppresses CS-induced EMT in mouse lungs.

### JSLJD promoted CFTR expression via the suppression of TGF-β/Smad2 signaling

**Fig. 3:**
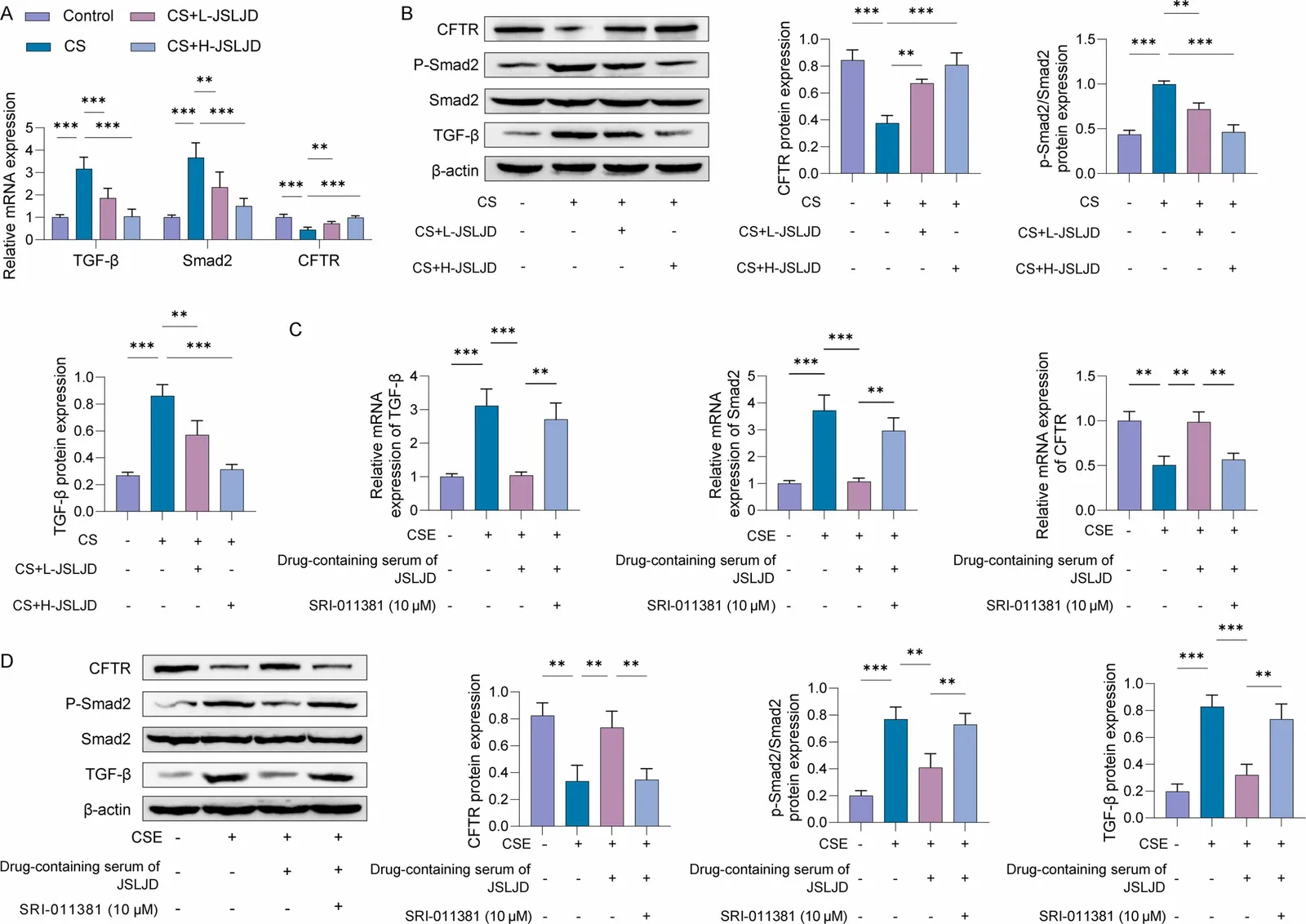
JSLJD promoted CFTR expression by inhibiting TGF-β/Smad2 signaling. (A) qPCR analysis of TGF-β, Smad2, and CFTR mRNA expression in mouse lungs. (B) Western blot detection of TGF-β, p-Smad2, Smad2, and CFTR in mouse lungs. (C) qPCR analysis of TGF-β, Smad2, and CFTR expression in 16HBE cells. (D) Western blot analysis of the expressions of the TGF-β, p-Smad2, Smad2, and CFTR proteins in 16HBE cells. Data are presented as the mean ± SD (n = 6 mice per group for animal experiments; n = 3 independent experiments for cell experiments). Statistical comparisons among the Control, CS, CS + L-JSLJD, and CS + H-JSLJD groups in the animal experiments and among the Control, CSE, CSE + JSLJD-containing serum, and CSE + JSLJD-containing serum + SRI−011381 groups in the cell experiments were performed using a one-way ANOVA followed by Tukey’s multiple comparisons test. **P < 0.01 and ***P < 0.001 indicate significant differences between the indicated groups.

Given that the TGF-β/Smad2 pathway is a major mediator of EMT, we further investigated whether JSLJD exerts its effects by modulating this pathway. qPCR and Western blot analyses revealed that CS exposure activated the TGF-β/Smad2 signaling pathway, which was accompanied by elevated TGF-β and p-Smad2 expression and the concomitant downregulation of CFTR expression in mouse lung tissues. JSLJD treatment significantly inhibited TGF-β/Smad2 activation and restored CFTR expression (Fig. 3A–B). Similar results were observed in vitro, where the CSE treatment of 16HBE cells increased TGF-β/Smad2 activation and reduced CFTR expression. Treatment with JSLJD-containing serum restored CFTR levels and suppressed TGF-β/Smad2 signaling, whereas the addition of the TGF-β activator SRI−011381 reversed these effects (Fig. 3C–D). These results suggest that JSLJD promotes CFTR expression by suppressing TGF-β/Smad2 signaling both in vivo and in vitro.

### JSLJD inhibited airway epithelial cell EMT through the TGF-β/Smad2/CFTR axis

**Fig. 4:**
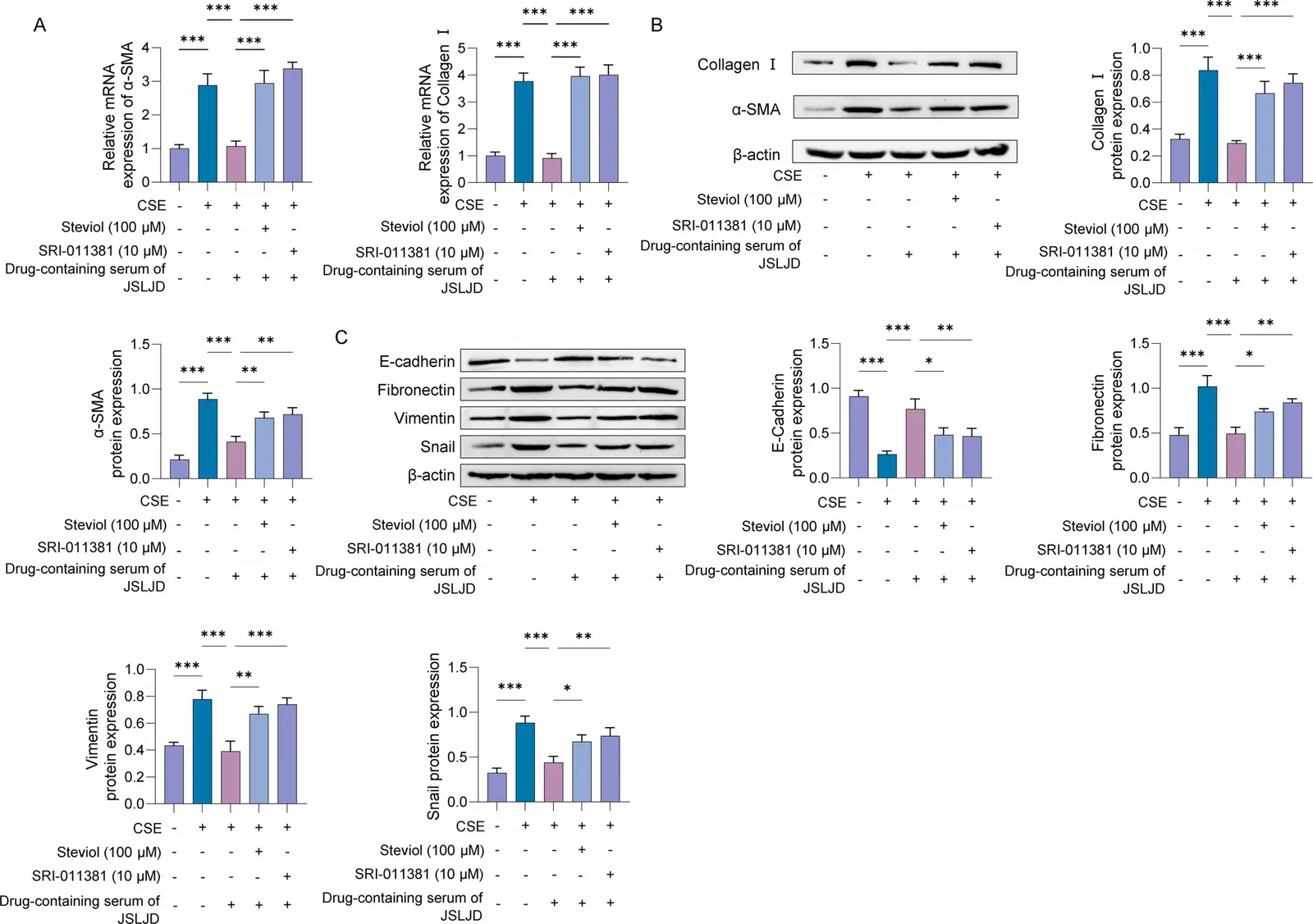
JSLJD inhibited bronchial epithelial EMT through the TGF-β/Smad2/CFTR axis. (A) qPCR detection of α-SMA and Collagen I mRNA expression in 16HBE cells. (B) Western blot detection of α-SMA and Collagen I protein expression in the same cells. (C) Western blot analysis of the expression of the EMT-related proteins E-cadherin, fibronectin, vimentin, and Snail. Data are presented as the mean ± SD from three independent experiments (n = 3 independent experiments). Statistical comparisons among the Control, CSE, CSE + JSLJD-containing serum, CSE + JSLJD-containing serum + Steviol, and CSE + JSLJD-containing serum + SRI−011381 groups were performed using a one-way ANOVA followed by Tukey’s multiple comparisons test. *P < 0.05, **P < 0.01, and ***P < 0.001 indicate significant differences between the indicated groups.

Finally, pharmacological interventions were conducted in a cell model of CSE-stimulated 16HBE cells to verify whether the inhibitory effect of JSLJD on airway epithelial cell EMT is achieved through the TGF-β/Smad2/CFTR axis. qPCR and Western blot analyses revealed that the inhibition of CFTR with steviol (100 µM) or the activation of TGF-β signaling with SRI−011381 (10 µM) reversed the anti-EMT effects of the JSLJD-containing serum, resulting in increased α-SMA and Collagen I expression (Fig. 4A–B). Moreover, under these conditions, mesenchymal markers, including fibronectin, vimentin, and Snail, were upregulated, whereas the epithelial marker E-cadherin was downregulated (Fig. 4C). Collectively, these results suggest that the anti-EMT activity of JSLJD may be mediated through the TGF-β/Smad2/CFTR signaling axis.

## Discussion

COPD is defined as irreversible airflow obstruction, with small airway remodeling acting as a major pathological driver of disease progression. Increasing evidence has demonstrated that EMT is a key contributor to airway structural alterations [19], [20], [21]. JSLJD is traditionally used to treat respiratory diseases, and it can significantly improve respiratory and immune functions in patients with COPD [22]. However, whether it can alleviate small airway remodeling in COPD by suppressing EMT has remained unclear. In the present study, JSLJD inhibited TGF-β/Smad2 signaling and restored CFTR expression, thereby suppressing EMT and ultimately alleviating CS-induced small airway remodeling.

Previous research has confirmed the therapeutic potential of JSLJD for pulmonary injury and airway pathology. Wang's research demonstrated that combining JSLJD with salmeterol/fluticasone for patients with stable COPD with lung and kidney deficiency can improve arterial blood gas indicators and airway inflammation, and it can help enhance lung function, thereby alleviating clinical symptoms [23]. Yue et al. reported that JSLJD treatment can effectively improve lung function in patients with chronic bronchitis and obstructive emphysema, reduce inflammatory responses, and be both effective and safe [24]. In our study, collagen accumulation and α-SMA expression were markedly reduced after the JSLJD intervention, suggesting the attenuation of peribronchiolar fibrosis. In addition, the suppression of EMT marker alterations—specifically, the reversal of E-cadherin loss and the downregulation of fibronectin, vimentin, and Snail—further supports the concept that JSLJD mitigates airway fibrosis by restoring epithelial homeostasis.

TGF-β/Smad signaling is excessively activated in COPD and serves as a major driver of epithelial injury, collagen deposition, and airway fibrosis. The inhibition of this pathway has been shown to attenuate structural remodeling and preserve epithelial integrity in COPD models [25]. Recent network pharmacology and transcriptomic analyses have further predicted that the therapeutic targets of JSLJD in COPD are enriched in genes related to TGF-β–related pathways and biological processes involving small airway remodeling [26]. In line with earlier results, the findings of the present study revealed that JSLJD markedly suppressed TGF-β/Smad2 activation in the lung tissues of smoke-exposed mice and in CSE-induced 16HBE cells, indicating that the inhibition of this profibrotic pathway represents a key mechanism underlying its protective effects.

CFTR dysfunction, a hallmark pathological change induced by CS exposure, leads to impaired mucociliary clearance, mucus blockage, and epithelial inflammation in patients with COPD [10]. Notably, emerging evidence indicates that reduced CFTR expression facilitates EMT and subsequent airway fibrosis. Our results revealed that CFTR expression was markedly reduced in both the CS-exposed mouse model and the CSE-treated 16HBE cells, whereas JSLJD treatment restored its expression. The pharmacological activation of TGF-β signaling suppressed the JSLJD-induced recovery of CFTR, suggesting that JSLJD enhances CFTR expression primarily through the inhibition of TGF-β/Smad2 signaling. Moreover, the reversal of the anti-EMT effects of JSLJD by TGF-β activation or CFTR inhibition confirms that the modulation of the TGF-β/Smad2/CFTR pathway is vital for preventing EMT progression.

The mechanistic interplay between TGF-β/Smad2 signaling and CFTR expression represents a novel axis connecting inflammation, EMT, and fibrosis. Mutant or deficient CFTR enhances TGF-β activity via TWIST1-dependent mechanisms, accelerating EMT progression [27], whereas the pharmacological restoration of CFTR expression has been reported to suppress TGF-β–induced fibrotic responses [17]. Our pharmacological rescue experiments reinforced this relationship: blocking CFTR or activating TGF-β signaling abolished the anti-EMT effects of JSLJD. These results extend the current understanding of JSLJD by identifying the TGF-β/Smad2/CFTR pathway as a convergent pathway through which it may coordinate the suppression of airway EMT and remodeling.

In summary, the present findings suggest that JSLJD inhibits TGF-β/Smad2 signaling and restores CFTR expression, thereby suppressing EMT and ultimately attenuating small airway remodeling. However, several limitations should be acknowledged. First, the current study was restricted to animal and cellular models. Second, the active compounds responsible for CFTR upregulation and TGF-β inhibition in JSLJD have yet to be identified. Third, additional signaling cascades, including NF-κB and PI3K/AKT, may participate in the multifaceted regulation of this process and warrant future exploration. Fourth, further multiomics and component-target analyses, combined with randomized clinical validation, are essential for clarifying the pharmacodynamic network of JSLJD and promoting its integration into evidence-based COPD therapeutics. Fifth, chemical fingerprinting and the quantitative determination of representative compounds were not performed in the present study. Future investigations incorporating systematic quality control approaches, such as voucher specimen preservation, batch traceability, high-performance liquid chromatography (HPLC) fingerprint analysis, and component-target validation, are warranted to further improve the standardization and reproducibility of JSLJD. Sixth, the JSLJD treatment was initiated concurrently with the cigarette smoke exposure, representing a preventive rather than a therapeutic intervention strategy. Although this design enabled the investigation of early protective effects and underlying mechanisms, it may not fully reflect clinical scenarios in which COPD is already established. Future studies using established COPD models with postonset JSLJD administration are warranted to further evaluate the therapeutic efficacy and translational potential of JSLJD. Another limitation of this study is the lack of pulmonary function measurements. Although the JSLJD treatment improved the histological features of airway remodeling and regulated EMT-related molecular changes, whether these structural and molecular improvements translate to functional respiratory benefits remains unclear. Future studies incorporating pulmonary function assessments, such as airway resistance and lung compliance measurements, are needed to comprehensively evaluate the therapeutic effects of JSLJD in patients with COPD. Finally, although the pharmacological activation and inhibition experiments supported the involvement of the TGF-β/Smad2/CFTR axis in mediating the effects of JSLJD, a direct functional assessment of CFTR activity and genetic manipulation approaches were not performed in the present study. Future studies using genetic approaches, such as CFTR knockdown or knockout models, combined with direct CFTR functional assays are warranted to further establish the causal role of CFTR in JSLJD-mediated airway protection.

## Conclusions

JSLJD inhibits TGF-β/Smad2 signaling and restores CFTR expression, thereby suppressing EMT and ultimately attenuating small airway remodeling.

## Ethics approval and consent to participate

All animal procedures were approved by the Institutional Animal Care and Use Committee of the Hunan Academy of Traditional Chinese Medicine Affiliated Hospital.

## Author contributions

Guo Ouyang and Wenjuan Xie guaranteed the integrity of the entire study. Wenjuan Xie and Qijun Zhou designed the study and literature research. Guo Ouyang and Yu Liu defined the intellectual content. Guo Ouyang and Qijun Zhou performed the experiments. Wenjuan Xie and Yu Liu collected the data. Qijun Zhou and Yu Liu analyzed the data. Guo Ouyang and Wenjuan Xie wrote the main manuscript and prepared the figures. All the authors reviewed the manuscript.

## Consent for publication

Not applicable

## Code availability

Not applicable

## Funding

This work was supported by the 10.13039/501100001809National Natural Science Foundation of China’s Young Scientists Fund (82004306), Natural Science Foundation of Hunan (2026JJ81883), and the Hunan Provincial Natural Science Foundation's Joint Medical and Health Project (2026JJ81856).

## CRediT authorship contribution statement

**Yu Liu:** Conceptualization, Data curation, Formal analysis, Writing – review & editing. **Guo Ouyang:** Writing – review & editing, Conceptualization, Project administration, Visualization, Writing – original draft. **Qijun Zhou:** Writing – review & editing, Visualization, Project administration, Formal analysis, Conceptualization. **Wenjuan Xie:** Writing – review & editing, Writing – original draft, Supervision, Data curation, Conceptualization.

## Declaration of Generative AI and AI-assisted technologies in the writing process

During the preparation of this work, the authors did not use any AI-assisted technology.

## Declaration of Competing Interest

The authors declare that they have no known competing financial interests or personal relationships that could have appeared to influence the work reported in this paper.

## Data Availability

The datasets used or analyzed during the current study are available from the corresponding author upon reasonable request.
